# Transient glyphosate encephalopathy due to a suicide attempt

**DOI:** 10.1002/npr2.12201

**Published:** 2021-08-06

**Authors:** Saaya Yokoyama, Tomomi Sugisaki, Yoshida Ryota, Sato Yoshiteru, Hiroaki Okayasu, Mukuto Shioda, Keisuke Suzuki, Norio Yasui‐Furukori, Kazutaka Shimoda

**Affiliations:** ^1^ Department of Psychiatry Dokkyo Medical University School of Medicine Shimotsuga Japan; ^2^ Department of Clinical Training Center Dokkyo Medical University School of Medicine Shimotsuga Japan; ^3^ Department of Neurology Dokkyo Medical University School of Medicine Shimotsuga Japan

**Keywords:** encephalopathy, glyphosate, MRI, suicide attempt

## Abstract

**Aim:**

Glyphosate‐containing herbicides are less toxic than paraquat and are one of the most widely used herbicides today.

**Case presentation:**

We report a case of ingestion of a pesticide containing glyphosate in a suicide attempt. The patient was admitted to the psychiatric department because of persistent suicidal thoughts. He suffered from short‐term memory impairment on day 3. Magnetic resonance imaging (MRI) showed swelling in the bilateral medial temporal lobes and hippocampi and high signal on T2‐weighted images. Gradually, the patient's cognitive impairments improved, and he was discharged on day 33.

**Conclusion:**

A physician should examine the patient with the possibility of glyphosate encephalopathy in mind.

## Abstract 2

The patient presented with delayed and transient short‐term memory impairment due to glyphosate medication. MRI showed bilateral medial temporal lobe and hippocampal swelling and high signal on T2‐weighted images, high signal in the right predominant insular cortex on diffusion‐weighted images, and high signal in the hippocampus and insular cortex on T2FLAIR.

## Graphical abstract

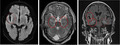

## 1 BACKGROUND

Glyphosate‐containing herbicides are less toxic than paraquat and are one of the most widely used classes of herbicides today. Glyphosate‐containing preparations are marketed at home improvement stores as safe herbicides. Therefore, with the increasing quantity of herbicides shipped, increasingly, many poisoning cases have occurred due to large doses of these compounds, and deaths have been reported. Gastrointestinal symptoms were the main symptoms of glyphosate poisoning. In severe cases, shock, acute renal failure, and disturbance of consciousness are observed.^1^, ^2^ Because of the ready availability of glyphosate‐containing herbicides, serious acute poisoning with these herbicides due to suicide attempts is a frequent occurrence, especially in developing countries and rural areas.

We report a case of transient encephalopathy after poisoning with a pesticide containing glyphosate as a suicide attempt. Written consent for publication was obtained from the patient.

## 2 CASE PRESENTATION

A 71‐year‐old man with no history of episodes suggesting mental illness was brought to us after a suicide attempt. Forty days earlier, he had undergone cataract surgery, and during the operation, the artificial lens fell inside the lens, resulting in a decrease in visual function compared with before the illness. Thereafter, he tended to have insomnia due to stress caused by the difficulty he faced in returning to his work and hobbies. He gradually developed depression, along with anxiety related to physical ailments such as discomfort in his right eye, back pain, and complaints of right knee pain. The depressive mood continued to worsen, and the patient complained that "I'm finished" and "I want to die"; he then ingested an herbicide (glyphosate) and was rushed to the emergency room. After gastric lavage, there was no physical abnormality, but as the patient's suicidal thoughts continued, the need for psychiatric treatment was recognized, and he was admitted to the psychiatric department the next day.

**FIGURE 1:**
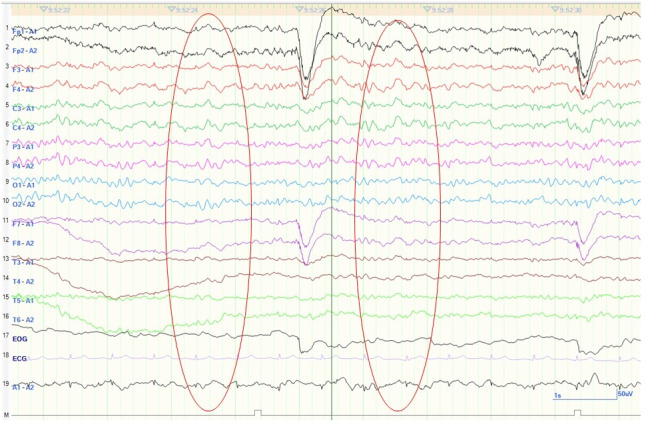
Electroencephalography (EEG) of the patient. EEG showed some 6‐7 Hz slow waves on the 12th day

**FIGURE 2:**
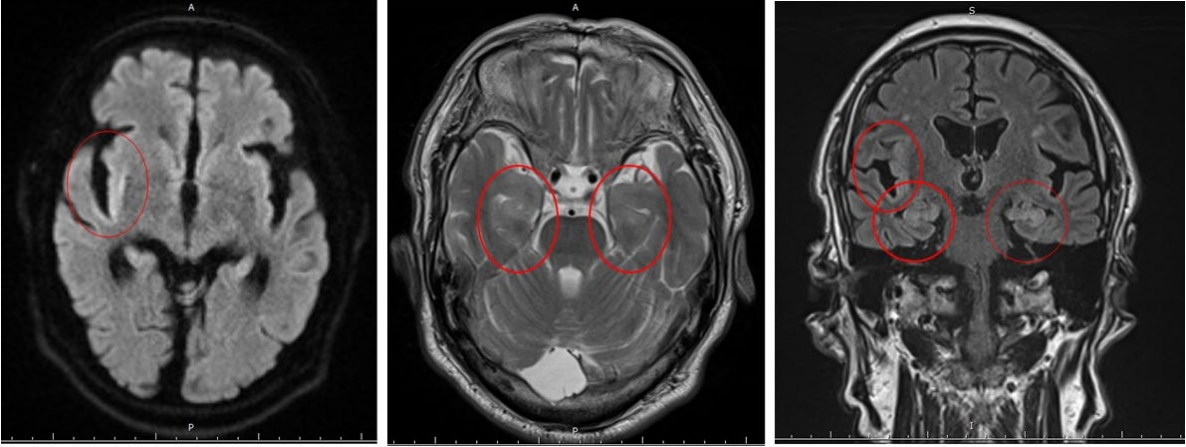
Diffusion‐weighted (left), T2 (middle), and T2‐FLAIR (right) MRI. MRI showed bilateral swelling of the medial temporal lobes and hippocampi and high signal on T2‐weighted imaging; diffusion‐weighted imaging showed high signal predominantly in the right insular cortex; and T2‐FLAIR also showed high signal predominantly in the right insular cortex. In addition to the insular cortex, T2‐FLAIR also showed a high signal in the hippocampus

The patient had been experiencing insomnia, depressed mood, weight loss of 5 kg in 1 month due to loss of appetite, poor concentration, low energy, feelings of worthlessness, loss of interest, and hopelessness for more than 2 weeks, triggered by the loss of vision caused by the surgery, which had made it difficult for him to lead his daily life without getting help from his family. Therefore, we diagnosed the patient with major depressive disorder and started him on mirtazapine (15 mg/day). On the seventh day, mirtazapine was discontinued due to suspected mirtazapine‐induced delirium. Although his anxiety and agitation improved slightly, the patient scored 23/30 points on the Hasegawa Dementia Scale‐Revised (HDS‐R), with some disorientation and short‐term memory impairment. The patient did not remember his suicide attempt or cataract surgery and had both anterograde and retrograde amnesia; on that basis, we suspected some organic cause. EEG showed some slow waves at 6‐7 Hz on the 12th day (Figure 1). MRI showed bilateral swelling of the medial temporal lobes and hippocampi and high signal on T2‐weighted imaging; diffusion‐weighted imaging showed high signal predominantly in the right insular cortex, and T2‐weighted fluid‐attenuated inversion recovery (T2‐FLAIR) also showed high signal predominantly in the right insular cortex (Figure 2). In addition to the insular cortex, T2‐FLAIR also showed a high signal in the hippocampus (Figure 2). On the 13th day, involuntary movements of the left face, tongue biting, and weakness of the right side of the body were observed with no impairment of consciousness; these movements were judged to be a simple partial seizure. On the same day, the patient was placed in the ICU for general management. There were no abnormal findings on a CT scan of the head. The patient was given 1000 mg of levetiracetam. On the following day, he was free of convulsions and hemiplegia and was able to communicate well; therefore, he returned to the psychiatric ward that day. On the 14th day, the patient returned to the psychiatric ward. An electroencephalogram (EEG) showed spikes in the right frontal lobe (F4), suggesting epilepsy originating in this region, and levetiracetam 1000 mg was continued. A lumbar puncture performed on the same day showed no increase in cerebrospinal fluid cell count, and cultures were negative, suggesting encephalopathy rather than infectious or autoimmune encephalitis. Gradually, in addition to the original disorientation, the patient became agitated, had difficulty maintaining attention, and was unable to receive rest instructions. For this reason, we considered him to be in a state of delirium and started physical restraint on the same day. At night, the patient became emotionally unstable, shouting, "I'm going to the office," yelling, and suddenly bursting into tears. In addition, from the 15th to the 16th day, 11 bouts of involuntary movements occurred on the left side of the face, lasting a few minutes each. The dose of levetiracetam was increased to 2000 mg/day on the 16th day, and simple partial epileptic seizures did not occur from then on. Risperidone (0.5 mg/day) was added before bedtime on the 17th day, and although there were some fluctuations in affect and disorientation after that, the patient was able to sleep at night and received rest instructions on the 19th day. Risperidone was discontinued. Gradually, the patient recovered from his cognitive impairments, including disorientation, and the HDS‐R on the 26th day showed a score of 23/30, with improvement only in the recall item. An EEG on the 27th day showed no slow waves or spikes, and an MRI scan on the 28th day showed a slight improvement in the high signal and swelling of the hippocampus and insular cortex on T2‐weighted, T2‐FLAIR, and diffusion‐weighted imaging; on this basis, the patient was discharged on the 33rd day.

## 3 DISCUSSION AND CONCLUSION

We reported a case of encephalopathy caused by glyphosate. In general, glyphosate poisoning is known to cause gastrointestinal symptoms and hepatic and renal dysfunction, and severe cases require ventilator management and hemodialysis. Consistent with the limited number of case reports, the following findings were noted: (a) delayed onset of short‐term memory impairment 2 days after oral administration; (b) high signal and swelling of the hippocampus on T2 MRI, T2‐FLAIR, and diffusion‐weighted imaging; and (c) high levels of protein in the cerebrospinal fluid, which supported the usefulness of these findings for the diagnosis of glyphosate encephalopathy.^3^, ^4^

Cattani et al reported that glyphosate may cause glutamate excitotoxicity and oxidative stress‐induced cell death in the rat hippocampus.^5^ According to Sedlaczek et al, the CA1 region, located in the perfusion zone of the posterior cerebral artery, has been identified as the most vulnerable hippocampal region to ischemia and is associated with transient global amnesia‐like memory impairment. In addition, they reported that hippocampal blood flow reduction due to stress, vasoconstriction, migraine, venous congestion, etc, is a factor in transient global amnesia.^6^ In addition, Yamana et al reported that left posterior aortic infarction with left medial temporal lobe lesion is associated with amnesia syndrome, and medial temporal lobe lesion is associated with pure dyslexia or dyslexia with dyscalculia.^7^ Powell et al noted that memory, unlike language function, is not dominant; one hemisphere or the other is sufficient, and in the case of unilateral lesions, the other side of the limbic system compensates, indicating that the lesions are transient and capable for the improvement.^8^

In the present case, the sudden onset of disorientation and "severe confusion, repeatedly asking the same questions about the date and the surrounding environment" closely resembled the symptoms of transient global amnesia, except that it was irreversible. Single‐photon emission computed tomography (SPECT) showed decreased blood flow in the dominant hemisphere (the patient was right‐handed), including the left frontal lobe, temporal lobe, bilateral parietal lobes, and left occipital lobe; the decreased blood flow in the left parietal lobe was particularly noticeable.

One limitation is that, although the patient's HDS‐R score improved, several components of his recovery—including the improvement of depression triggered by the onset of encephalopathy, the control of symptomatic epilepsy, and the improvement of cognition—could have contributed; it is difficult to distinguish the causal relationships of these variables. In addition, we cannot rule out the possibility that the decrease in cerebral blood flow shown by SPECT was due to a chronic decrease in blood flow caused by hypertension, alcohol consumption, and smoking. However, at least in this case, the patient had been working as a truck driver and an office worker until 1 month before the suicide attempt, and he had shown no loss of short‐term memory or of his ability to write or perform arithmetic.

In this case, the glutamate excitotoxicity and oxidative stress caused by glyphosate may have caused a decrease in blood flow not only to the posterior cerebral artery, which is the dominant artery supplying the hippocampus, but also to the middle cerebral artery, which perfuses the parietal lobe. In addition, in this case, the left hemisphere, which is the dominant hemisphere, was predominantly hypoperfused, and there was a gradual tendency toward memory impairment, although verbal learning remained intact. This suggests that SPECT may be effective in predicting the prognosis of memory impairment by evaluating whether the blood flow decrease is unilateral or bilateral. Assuming that the causative pathology is vascular, risk factors for glyphosate encephalopathy include advanced age, hypertension, hyperlipidemia, diabetes mellitus, and a history of drinking or smoking. Three of the four reported cases of memory impairment with glyphosate poisoning, including hippocampal infarction, were in patients over 60 years old.^3^, ^5^, ^9^ The present patient was 71 years old, had smoked 25 cigarettes/day for more than 20 years, had been drinking 3 cups of sake/day for the past month, and had controlled hypertension.

Glyphosate is not known to cause encephalopathy, and asymptomatic or mild cases may not prompt MRIs or other imaging studies; such cases may be diagnosed only as part of the symptoms of depression or delirium or may be misdiagnosed as a dissociative disorder. Therefore, when we encounter an emergency case of glyphosate poisoning, we should examine the patient with the possibility of glyphosate encephalopathy in mind, which may guide future treatment and help to further elucidate the pathogenesis of glyphosate encephalopathy.

## CONFLICT OF INTEREST

The authors declare that they have no competing interests.

## AUTHOR CONTRIBUTIONS

SY, ST, RY, and MS treated the patient and acquired data. NYF wrote the manuscript and analyzed image data. SY drafted the work, analyzed image data. HO, KS, and KS supervised the work and substantively revised it. All authors read and approved the final manuscript.

## APPROVAL OF THE RESEARCH PROTOCOL BY AN INSTITUTIONAL REVIEWER BOARD

The ethics committee is not required to review case reports.

## INFORMED CONSENT

The patient has consented in a written form to the submission of the case report for submission to the journal.

## Data Availability

The data are not publicly available due to privacy restrictions.
